# Rational design of an acidic erythritol (ACER) medium for the enhanced isolation of the environmental pathogen *Burkholderia pseudomallei* from soil samples

**DOI:** 10.3389/fmicb.2023.1213818

**Published:** 2023-06-30

**Authors:** Karoline Assig, Sabine Lichtenegger, Linh N. H. Bui, Bettina Mosbacher, Anh T. N. Vu, Daniel Erhart, Trung T. Trinh, Ivo Steinmetz

**Affiliations:** ^1^Diagnostic and Research Institute of Hygiene, Microbiology and Environmental Medicine, Medical University Graz, Graz, Austria; ^2^Institute of Microbiology and Biotechnology, Vietnam National University, Hanoi, Vietnam

**Keywords:** *Burkholderia pseudomallei*, environment, detection, soil, culture medium

## Abstract

The soil bacterium *Burkholderia pseudomallei* causes melioidosis, a potentially fatal and greatly underdiagnosed tropical disease. Detection of *B. pseudomallei* in the environment is important to trace the source of infections, define risk areas for melioidosis and increase the clinical awareness. Although *B. pseudomallei* polymerase chain reaction (PCR)-based environmental detection provides important information, the culture of the pathogen remains essential but is still a methodological challenge. *B. pseudomallei* can catabolize erythritol, a metabolic pathway, which is otherwise rarely encountered among bacteria. We recently demonstrated that replacing threonine with erythritol as a single carbon source in the pH-neutral threonine-basal salt solution (TBSS-C50) historically used improved the isolation of *B. pseudomallei* from rice paddy soils. However, further culture medium parameters for an optimized recovery of *B. pseudomallei* strains from soils are still ill-defined. We, therefore, aimed to design a new erythritol-based medium by systematically optimizing parameters such as pH, buffer capacity, salt and nutrient composition. A key finding of our study is the enhanced erythritol-based growth of *B. pseudomallei* under acidic medium conditions. Our experiments with *B. pseudomallei* strains from different geographical origin led to the development of a phosphate-buffered acidic erythritol (ACER) medium with a pH of 6.3, higher erythritol concentration of 1.2%, supplemented vitamins and nitrate. This highly selective medium composition shortened the lag phase of *B. pseudomallei* cultures and greatly increased growth densities compared to TBSS-C50 and TBSS-C50-based erythritol medium. The ACER medium led to the highest enrichments of *B. pseudomallei* as determined from culture supernatants by quantitative PCR in a comparative validation with soil samples from the central part of Vietnam. Consequently, the median recovery of *B. pseudomallei* colony forming units on Ashdown’s agar from ACER subcultures was 5.4 times higher compared to TBSS-C50-based erythritol medium (*p* = 0.005) and 30.7 times higher than TBSS-C50 (*p* < 0.001). In conclusion, our newly developed ACER medium significantly improves the isolation of viable *B. pseudomallei* from soils and, thereby, has the potential to reduce the rate of false-negative environmental cultures in melioidosis risk areas.

## Introduction

Melioidosis is a massively underdiagnosed infectious disease caused by the soil bacterium *Burkholderia pseudomallei*. Estimates suggest the occurrence of 165,000 melioidosis cases per year in tropical and subtropical countries with approximately 89,000 fatalities (Limmathurotsakul et al., 2016). There is currently no vaccine available. Based on environmental suitability *B. pseudomallei* is likely to exist in many parts of the world where neither melioidosis cases have been diagnosed nor the bacterium detected in the environment so far. Since *B. pseudomallei* is not evenly distributed in the environment of endemic areas and its occurrence depends on multiple variables, such as rainfall, temperature, soil type, and anthropogenic activities (Limmathurotsakul et al., 2010, 2016), environmental surveillance is of particular importance. The environmental detection of *B. pseudomallei* can be a crucial hint for the existence of melioidosis and starting to search for *B. pseudomallei* in clinical specimens. Although *B. pseudomallei* DNA detection in soil and water provides important information on the environmental presence, cultural isolation of the pathogen remains indispensable for studying its antibiotic resistance, virulence and population structure, as well as tracing back sources of infection. The significantly higher rate of polymerase chain reaction (PCR)-based *B. pseudomallei* detection performed from environmental samples compared to culture-based methods (Knappik et al., 2015; Gohler et al., 2017) strongly suggests significant numbers of false-negative culture results. The environmental detection of *B. pseudomallei* from soil in the past has commonly relied on an enrichment in the currently recommended threonine-basal salt solution with colistin (TBSS-C50), followed by subculture on Ashdown agar (Limmathurotsakul et al., 2013). The insufficient sensitivity of this approach results, at least partly, from inadequate selectivity, since related bacteria in soil samples with similar growth and antibiotic resistance characteristics compete with *B. pseudomallei* during enrichment and subculture (Wuthiekanun et al., 1995; Walsh and Duffy, 2013).

In order to address the high rate of false-negative environmental cultures, we recently improved the selectivity of TBSS-C50 by replacing nitrilotriacetic acid with NH_4_H_2_PO_4_ and threonine with erythritol as a single carbon source. This modification was based on the fact that erythritol catabolism is not widespread among bacteria and seems to be a unique feature of *B. pseudomallei*, which has not been seen in related *Burkholderia* spp. so far (Yabuuchi et al., 1992). We found that a two-step enrichment of rice paddy soil samples in TBSS-C50 for 48 h medium, followed by 96 h incubation in TBSS-C50-based erythritol medium resulted in a six times higher yield of *B. pseudomallei* culture positive samples in comparison to the consensus guideline culture with TBSS-C50 for 48 h. The two-step enrichment with TBSS-C50-based erythritol medium resulted in about twice as much positive samples compared to a two-step culture with only TBSS-C50 medium (Trinh et al., 2019). Despite that improvement, no *B. pseudomallei* strains could be isolated in approximately 40% of PCR-positive enrichment samples, indicating still a potentially high number of false-negative culture results (Trinh et al., 2019).

In this study, we aimed to systematically optimize *B. pseudomallei* selective growth conditions in medium containing erythritol as a single carbon source. Experiments were performed to identify the optimum medium conditions for *B. pseudomallei* regarding pH, erythritol concentration, vitamin demand, salt content and nitrate, the latter being a potential alternative electron acceptor under low oxygen conditions. Our new chemically defined, *ac*idic *er*ythritol (ACER) medium exhibited significantly enhanced selective growth support for *B. pseudomallei*. In a comparative validation, ACER medium led to the highest enrichment of *B. pseudomallei* from soils and, finally, the highest recovery of viable *B. pseudomallei*.

## Materials and methods

### Bacterial strains

*Burkholderia pseudomallei* strains and other bacterial isolates used in this study are listed in Table 1.

**Table 1:** Bacterial strains.

Strain	Species	Source and origin
K96243	*Burkholderia pseudomallei*	Human, Thailand
E8	*B. pseudomallei*	Soil, Thailand
E212	*B. pseudomallei*	Soil, Thailand
NCTC7383	*B. pseudomallei*	Human, Burma
770,429	*B. pseudomallei*	Soil, Niger
MK441	*B. pseudomallei*	Monkey, Philippines
NCTC1688	*B. pseudomallei*	Rat, Malaysia
NCTC10276	*B. pseudomallei*	Human, Bangladesh
E27	*B. thailandensis*	Soil, Thailand
E232	*B. thailandensis*	Soil, Thailand
E264	*B. thailandensis*	Soil, Thailand
K56-2	*B. cenocepacia*	Human, Canada
LMG14291	*B. stabilis*	Human, Belgium
R-15281	*B. multivorans*	Human, Germany
LMG6889	*B. cepacia*	Not available
DSM17292	*Cupriavidus gilardii*	Whirlpool, United States

### Preparation of culture media

Threonine-basal salt solution with colistin (TBSS-C50) (Galimand and Dodin, 1982) and TBSS-C50-based erythritol medium (Trinh et al., 2019) were prepared as described by Limmathurotsakul and colleagues (Limmathurotsakul et al., 2013) and by Trinh and colleagues (Trinh et al., 2019), respectively. Both media differ in nitrogen and carbon sources. 1.05 mM nitrilotriacetic acid and 50 mM L-threonine in TBSS-C50 was replaced by 18.7 mM NH_4_H_2_PO_4_ and 0.4% (32.75 mM) erythritol as the single carbon source in TBSS-C50-based erythritol medium.

One liter of ACER medium was prepared by dissolving the base components: firstly, the buffer substances 4.75 g (34.9 mM) KH_2_PO_4_, 2.63 g (15.1 mM) K_2_HPO_4_ followed by 5 g (0.5%) NaCl were solubilized thoroughly in about 600 mL of destilled water. Following this, 0.12 g (0.49 mM) MgSO_4_*7H_2_O and 0.02 g (0.14 mM) CaCl_2_*2H_2_O, each diluted in 50 mL distilled water beforehand, were added to the solution. In the next step, 2.15 g (18.7 mM) NH_4_H_2_PO_4_ and 10 mL of a 1 M NaNO₃ stock (final 10 mM) were added. The base was adjusted to pH 6.3, filled up with distilled water to 876 mL and either sterile filtered or autoclaved. After sterilization, the base was combined with 100 mL sterile filtered erythritol solution of 12% (final 1.2% per liter) and 4 mL of an autoclaved micro salt solution (see below). One liter of the micro salt solution stock contained 11.53 mL H_3_PO_4_ 85%, 1.49 g ZnSO_4_*7H_2_O, 0.11 g CuSO_4_*5H_2_O, 0.63 g MnSO_4_*H_2_O, 0.15 g Co(NO_3_)_2_*6H_2_O, 0.15 g Na_2_MoO_4_*2H_2_O, and 0.31 g H_3_BO_3_ in 1000 mL distilled water. To prevent precipitation, 400 μL of 100 mM FeSO_4_*7H_2_O solved in 2 M HCl and stored at −20°C was added separately per liter of ACER broth (final working concentration 40 μM) directly before use. Also prior to experiments, 20 mL 100x Gibco^™^ MEM Vitamin Solution, containing 170 mg NaCl, 2 mg D-Calcium pantothenate, 2 mg choline chloride, 2 mg folic acid, 4 mg i-inositol, 2 mg nicotinamide, 2 mg pyridoxine · HCl, 0.2 mg riboflavin and 2 mg thiamine · HCl, was added. Finally, the colistin sulfate concentration (Carl Roth, Austria) was adjusted to 50 mg/L. Cyclohexmide was also added at a final concentration of 50 mg/L for soil culture experiments. All of the salts and reagents were purchased from either Sigma Aldrich (Austria) or Carl Roth (Austria) suppliers.

Ashdown agar was prepared as described (Ashdown, 1979) containing 10 g trypticase soy broth (Becton Dickinson, United States), 15 g agar, 40 mL 98% glycerol (Fisher Chemical, United Kingdom), 5 mg crystal violet (Sigma-Aldrich, India), 50 mg neutral red (Sigma-Aldrich, United States) supplemented with 5 mg gentamicin (Carl Roth, Germany) per liter.

### Bacterial growth experiments

Regarding growth experiments in liquid culture, bacteria were initially grown on Columbia agar containing 5% sheep blood (BD Biosciences, Austria) at 37°C for 20 h under aerobic conditions. Bacteria were harvested from blood plates and washed twice with phosphate buffered saline (PBS) at 6000 g for 2 min. Cultures were inoculated to an initial optical density of 0.01 at 600 nm (OD_600_). Culture experiments were performed at 40°C either in a volume of 200 μL medium in a Bioscreen C instrument (Labsystems, Helsinki, Finland) under continuous shaking, in 50 mL falcon tubes containing a volume of 10 mL medium or in 125 mL flasks filled with 25 mL of the respective medium. Cultures in falcons and flasks were either shaken at 120 rpm or incubated statically. OD_600_ measurements were performed at the indicated time points. Measurements of pH of bacterial cultures were performed using the HANNA instrument HI98103 (Hanna Instruments Austria).

### Soil samples

**Figure 1:**
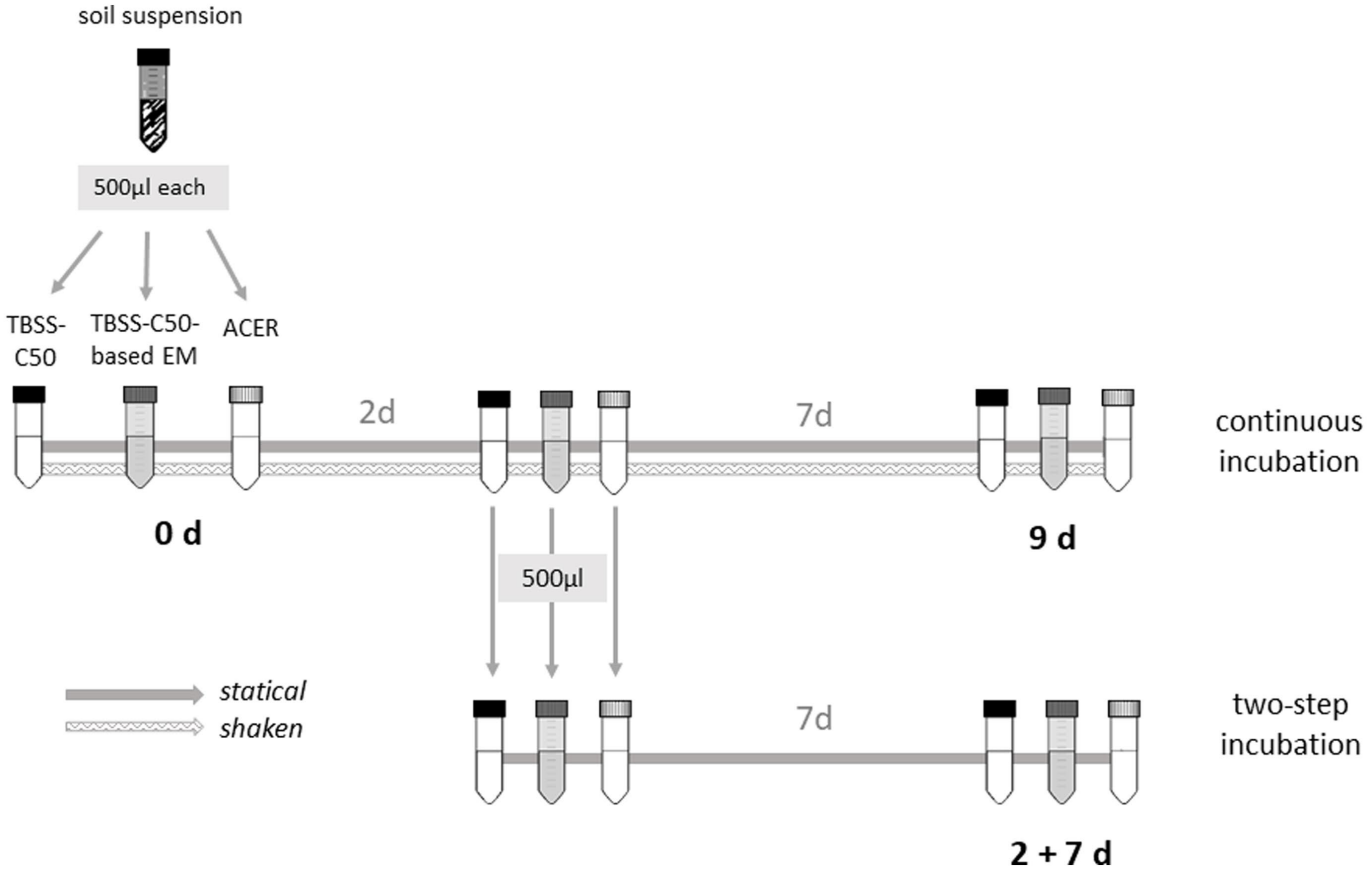
Schematic overview of the experimental setup. Twenty soil samples collected in Vietnam in October 2022 were enriched in ACER, TBSS-C50-based erythritol medium and TBSS-C50 in a single incubation step for 9  days, shaken and statically, and in a 2-step incubation for 2  days followed by 7  days under static conditions. After 9  days, 500 μL aliquots of each culture were taken for *B. pseudomallei*-specific qPCR and for cultivation on Ashdown agar. Samples were preserved either at −80°C as cell pellets for qPCR analyses or as glycerol stocks of 25% glycerol for cultivation.

Twenty soil samples were collected at the end of the wet season in October 2022 in the north-central part of Vietnam from two adjacent sites, a sugar cane field and an uncultivated field separated by a road. These sites were chosen because a previous environmental culture screening in 2017 revealed a high number of *B. pseudomallei* culture-positive samples in the sugar cane field (Lichtenegger et al., 2021). All soil samples were taken at a depth of 30 cm, with a distance of 5 to 10 m between each other. The steel auger used for collection was cleaned with bottled water and disinfected with 70% alcohol after each sampling point. Approximately 200 g of soil samples collected in plastic zip bags were kept at an ambient temperature and transferred immediately to the Institute of Microbiology and Biotechnology, Vietnam National University, Hanoi. Samples were divided equally and shipped to the Institute of Hygiene, Microbiology and Environmental Medicine, Medical University of Graz, Austria at ambient temperature. Upon arrival in Graz, the soil samples were kept in sealed plastic bags at room temperature and subsequently used for soil enrichment culture experiments (Figure 1). The content of clay, silt and sand was quantified by the pipette method and soil texture was classified by referencing the textural triangle (Staff, 2017) at the Soils and Fertilizers Research Institute (SFRI), Vietnam Academy of Agricultural Sciences. Soil samples were categorized as clay loam (*n* = 2) and loam (*n* = 18).

### Soil enrichment cultures

5 g of soil from each sample was homogenized and dissolved in 10 mL PBS for the comparative culture protocol validation. The soil-PBS suspensions were shaken overnight with closed lids at 160 rpm at room temperature. After sedimentation for about 20 min, the upper aqueous phase was collected in fresh tubes and thoroughly mixed. A volume of 500 μL of the aqueous soil suspension was added to 10 mL of ACER, TBSS-C50-based erythritol medium and TBSS-C50 for inoculation. Another 500 μL soil suspension aliquot was kept for PCR analysis and centrifuged for 10 min at 18,213 g, the supernatant discarded and the pellet frozen at −80°C until use. Cultures were incubated with and without shaking at 120 rpm at 40°C for 9 days. Shaken cultures were incubated with closed lids, static cultures with loose caps. Following 48 h of incubation, additional sub-enrichments of all media were started: A volume of 500 μL of the 48 h enrichments was transferred to 10 mL of the respective fresh medium and further incubated statically for 7 days (Figure 1). A volume of 500 μL of the enrichment cultures was frozen on the last day of each enrichment culture either as a pellet, as described above for qPCR analysis, or as glycerol stock (25% glycerol) at −80°C for subsequent subcultures on agar.

### Bacterial DNA extraction from soil cultures and determination of *Burkholderia pseudomallei* load by TTSS1 quantitative PCR

The DNA extraction from bacterial pellets was performed by using the NucleoSpin^®^ Microbial DNA Kit from Macherey-Nagel with slight modifications as described previously (Wagner et al., 2023). Quantitative PCR was performed using a probe-based PCR real-time assay specific for a TTSS1 gene region of *B. pseudomallei* (Novak et al., 2006; Trinh et al., 2019).

### Determination of bacterial counts from soil cultures

Glycerol stocks of soil enrichment cultures were thawed, mixed thoroughly and 100 μL of tenfold serial dilutions plated in duplicate on Ashdown agar. Plates were incubated at 40°C for 4 days followed by 1 day at room temperature. Morphologically suspicious *B. pseudomallei* colonies were identified and confirmed by PCR, targeting the *B. pseudomallei* specific sequence of the TTSS1 gene (Novak et al., 2006).

### Statistical analysis

Statistical analyses and calculations were performed using GraphPad Prism software version 9.2.0 (GraphPad Software, San Diego, CA, United States). The comparison between optical densities at respective time points and media compositions was done with the Wilcoxon matched-pairs signed rank test and Friedman test followed by Dunn’s multiple comparisons test. The growth of eight *B. pseudomallei* strains in ACER, TBSS-C50, and TBSS-C50-based erythritol media was analyzed with Fisher’s exact test. *C_T_* values and colony forming units (CFUs) of different enrichment cultures of the same soil sample suspension were analyzed as paired data with the nonparametric Friedman test followed by Dunn’s multiple comparisons test. Missing *C_T_* values were assigned to an arbitrary *C_T_* of 40 to account for the need of complete data sets for Friedman analysis. The interrelation between cultivated *B. pseudomallei* bacteria on Ashdown agar and respective *C_T_* values was calculated with the Spearman’s rank correlation. Median cycle threshold (*C_T_*) values of TTSS1-qPCR data from soil enrichments are indicated in the text in parentheses with the lower quartile q1 and the upper quartile q3.

## Results

### Early growth of *Burkholderia pseudomallei* on erythritol is increased under acidic conditions

**Figure 2:**
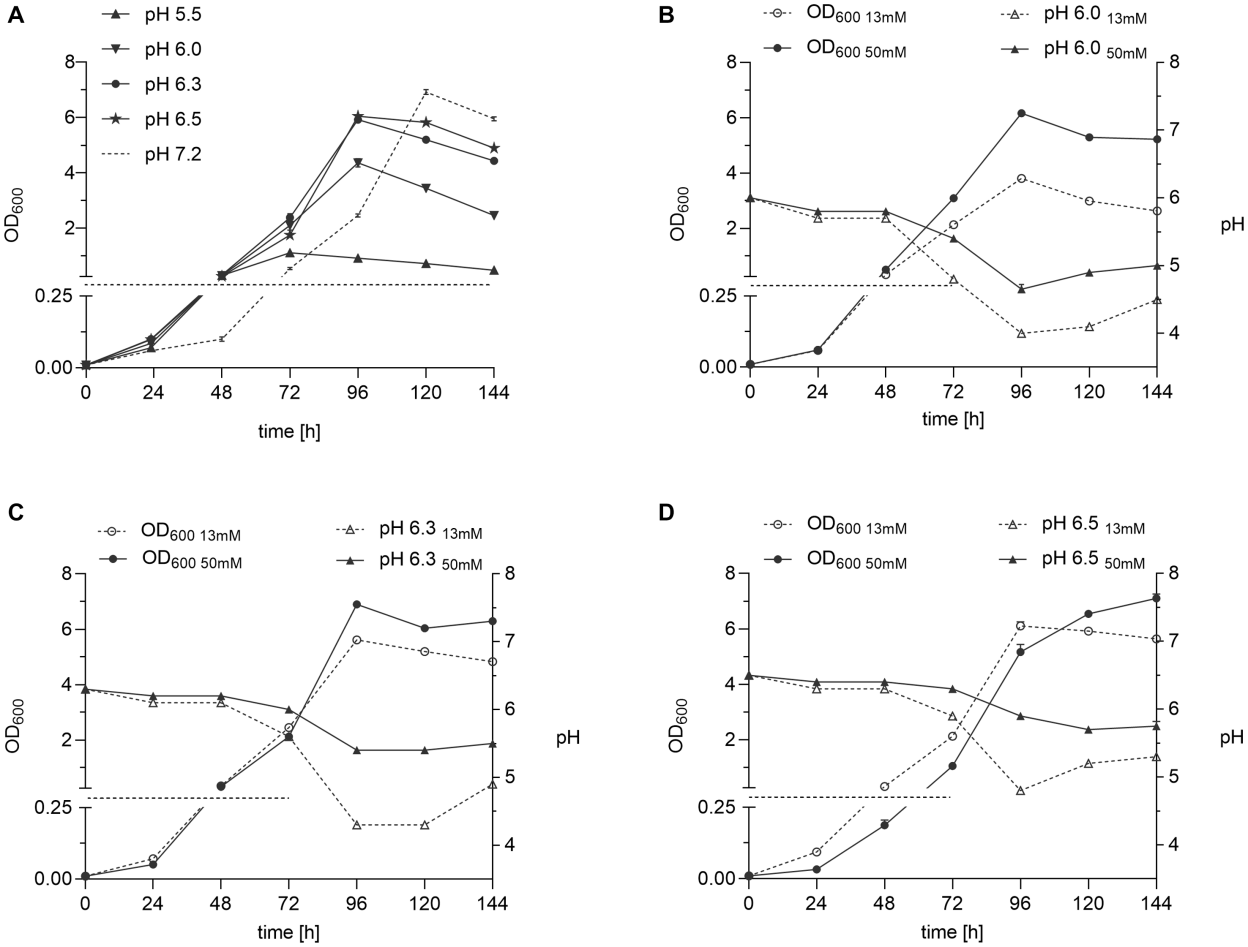
Effect of pH and buffer concentration on *B. pseudomallei* growth in erythritol medium. **(A)** Growth of strain K96243 in 25 mL of TBSS-C50-based erythritol medium adjusted to pH 5.5, 6.0, 6.3, 6.5, and 7.2 at 40°C and 120 rpm. **(B**–**D)** Cultures of *B. pseusomallei* strain K96243 grown with either 13 or 50 mM phosphate buffer at pH 6.0 **(B)**, 6.3 **(C)** and pH 7.2 **(D)**. OD_600_ data are plotted on the left axis (note the broken axis highlighted by a dashed line and different scales), pH values on the right axis. Growth curves are representative of at least two independent experiments, each experiment of which was conducted in technical duplicates. Error bars denote the standard deviation of mean from technical duplicates of a single experiment.

We recently demonstrated the highly selective growth of *B. pseudomallei* strains in TBSS-C50-based erythritol medium (Trinh et al., 2019). We aimed here to optimize medium parameters to further improve growth and, at the same time, preserve selectivity. Since there is evidence that the occurrence of the genus *Burkholderia* (Stopnisek et al., 2014) and particularly *B. pseudomallei* (Chen et al., 2003; Kaestli et al., 2007; Palasatien et al., 2008; Kaestli et al., 2009) is associated with acidic soil conditions, we, firstly, investigated the influence of the medium pH on the growth of various *B. pseudomallei* strains (Table 1) in a pH range from 5.5 to 7.2 (Figure 2A; Supplementary Figure S1).

All strains benefited in early growth during the first 24 h from an initial acidic medium pH down to 6.0 (Figure 2A; Supplementary Figures S1, S2). After 72 h of enrichment, we still saw a significant increase in growth for pH 6.3 compared to 6.0 and 6.5 (Supplementary Figure S2). However, we observed that in cultures of lower initial pH, this positive trend was reversed with ongoing incubation time (Figure 2A; Supplementary Figure S1). We hypothesized that this later growth stagnation of initially acidic cultures was caused by the continuing acidification of the culture. We increased the buffer capacity from 13 to 50 mM to maintain the advantage of a shortened lag phase but stabilize the growth in acidic culture medium over time. As depicted in Figures 2B–D, higher buffered cultures with an initial pH of 6.3 (Figure 2C) reached higher optical densities than cultures of pH 6.0 (Figure 2B) and 6.5 (Figure 2D) within 96 h and comparable optical densities at 144 h compared to pH 7.2 (Figure 2A). Therefore, a 50 mM phosphate buffer concentration and a pH of 6.3 were chosen for subsequent medium optimization experiments.

### Increased erythritol concentration and the addition of vitamins further improves *Burkholderia pseudomallei* growth

**Figure 3:**
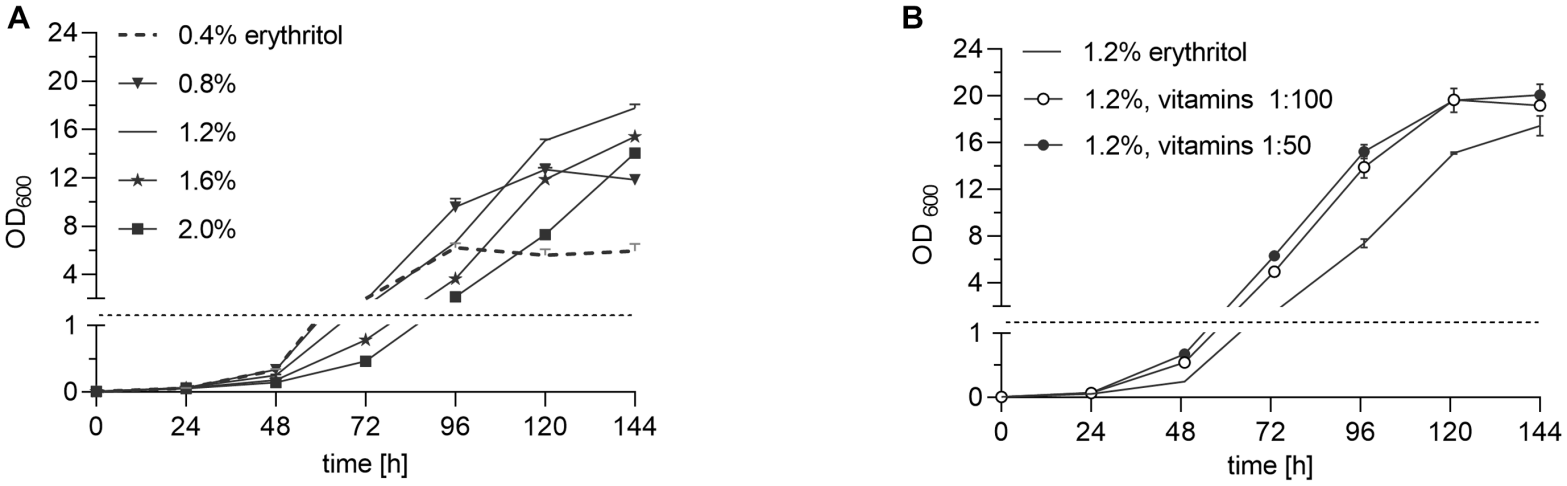
Effect of erythritol concentration and vitamins on *B. pseudomallei* growth. **(A)** *B. pseudomallei* K96243 cultivated in 25 mL TBSS-C50-based erythritol medium at pH 6.3 with 50 mM potassium phosphate buffer and varying erythritol concentrations (0.4, 0.8, 1.2, 1.6, and 2%) for 144 h at 40°C and 120 rpm. **(B)** *B. pseudomallei* K96243 cultivated in TBSS-C50-based erythritol medium at pH 6.3, 50 mM potassium phosphate buffer, with 1.2% erythritol and Gibco MEM Vitamin Solution diluted 1:100 and 1:50. Note the broken *y*-axis highlighted by a dashed line and different scales. Growth curves are representative of at least two independent experiments, each experiment of which was conducted in technical duplicates. Error bars denote the standard deviation of mean from technical duplicates of a single experiment.

We next tested different erythritol concentrations in 50 mM phosphate-buffered TBSS-C50-based erythritol medium at pH 6.3. Increasing the erythritol concentration up to 1.2% led to more than twice higher optical density compared to erythritol concentrations of 0.4% (Figure 3A), meanwhile a further increase in the erythritol concentration above 1.2% led to a reduction in growth. The lag phase was further reduced by the supplementation of vitamins (Gibco^™^ MEM Vitamin Solution). The optical densities had already more than doubled by day two of the incubation in media enriched with vitamins in 1:50 dilution, and on day 3 more than four-fold compared to growth in 1.2% erythritol medium without vitamins (Figure 3B). We therefore continued our medium optimization with 50 mM buffered medium at pH 6.3 containing 1.2% of erythritol and MEM vitamin solution diluted 1:50.

### Nitrate promotes *Burkholderia pseudomallei* growth with erythritol as the single carbon source under oxygen-limited conditions

**Figure 4:**
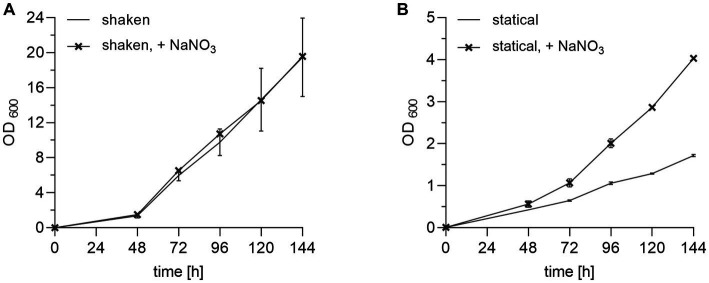
Effect of nitrate on growth of *B. pseudomallei* in erythritol medium. **(A)** *B. pseudomallei* strain K96243 cultivated under shaking conditions in 50 mL falcon tubes at 120 rpm in 10 mL TBSS-C50-based erythritol medium at pH 6.3, 50 mM potassium phosphate buffer, 1.2% erythritol, Gibco MEM Vitamin Solution diluted 1:50, with and without 10 mM nitrate for 144 h at 40°C. Nitrate addition is symbolized by crosses in the growth curves. **(B)** *B. pseudomallei* strain K96243 cultivated in the same media statically in 50 mL falcon tubes. Growth curves are representative of at least two independent experiments, each of which was conducted in technical duplicates. Error bars denote the standard deviation of mean from technical duplicates of a single experiment.

*B. pseudomallei* encounters hypoxic or even anaerobic conditions in its natural soil habitat (Tiedje et al., 1984; Liesack et al., 2000; Yamauchi et al., 2000; Angel et al., 2012). Therefore, at least a fraction of the soil-dwelling *B. pseudomallei* population might be metabolically adapted to a limited oxygen supply at the time when a soil sample is subjected to an enrichment culture. Furthermore, if static cultures are applied instead of shaken cultures, the availability of oxygen for cellular respiration is reduced. Since *B. pseudomallei* is capable of using nitrate as a terminal electron acceptor for respiration (Yabuuchi et al., 1992), we investigated the growth of *B. pseudomallei* strain K96243 in erythritol medium with the addition of nitrate. Prior to these experiments, the impact of different nitrate concentrations on *B. pseudomallei* shaken cultures was tested and 10 mM was chosen for subsequent experiments (Supplementary Figure S3). Figure 4 shows experiments with and without the addition of nitrate under shaken (Figure 4A) and static conditions (Figure 4B). Growth was generally clearly higher in shaken (Figure 4A) compared to static cultures (Figure 4B). Higher cell densities were observed in static enrichments in the presence of 10 mM sodium nitrate (Figure 4B). Since the addition of nitrate improved the growth under static culture conditions without any significant inhibitory effect, we included 10 mM nitrate in the medium formula from this point onwards.

Finally, we reduced the sodium chloride concentration in the medium base from 1 to 0.5% because some strains grew better at sodium chloride levels below 1% while none showed impaired growth at the reduced salt amount (Supplementary Figure S4). In summary, our newly composed *ac*idic *er*ythritol medium, designated ACER medium differs from our previous TBSS-C50-based erythritol medium in a lower pH of 6.3, an increased concentration of 50 mM phosphate buffer, an erythritol concentration raised to 1.2%, sodium chloride reduced to 0.5%, added vitamins in a dilution of 1:50 and the addition of 10 mM nitrate.

### Significantly improved *Burkholderia pseudomallei* growth in highly selective ACER medium compared to TBSS-C50-based erythritol medium and standard TBSS-C50

**Figure 5:**
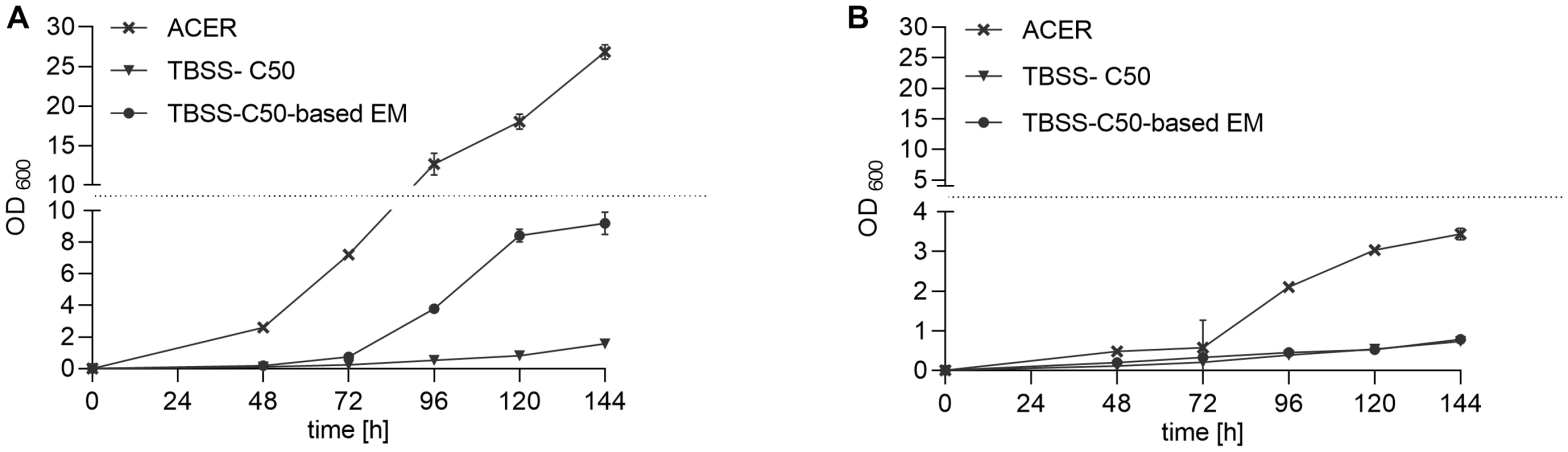
Growth of *B. pseudomallei* strain K96243 in ACER medium compared to TBSS-C50 and TBSS-C50-based erythritol medium (EM) under static and shaken conditions. **(A)** *B. pseudomallei* strain K96243 was cultivated in 10 mL ACER medium, TBSS-C50 and TBSS-C50-based erythritol medium, shaken at 120 rpm in in 50 mL falcons for 144 h at 40°C. **(B)** *B. pseudomallei* strain K96243 cultivated statically in 50 mL falcons for 144 h at 40°C in 10 mL of the respective media. Note the broken *y*-axis highlighted by a dashed line and different scales. Growth curves are representative of at least two independent experiments, each of which was conducted in technical duplicates. Error bars denote the standard deviation of mean from technical duplicates of a single experiment.

We, finally, validated the new ACER medium with eight *B. pseudomallei* strains (Figure 5; Supplementary Figures S5, S6) and compared their growth to TBSS-C50-based erythritol medium and TBSS-C50 under shaking (Figure 5A; Supplementary Figure S5) and static (Figure 5B; Supplementary Figure S6) conditions. After 144 h of shaking and static incubation, seven out of eight strains reached higher optical densities in ACER medium compared to TBSS-C50-based erythritol medium (*p* = 0.02) and all strains grew better compared to TBSS-C50 (*p* = 0.003). The selectivity of ACER medium was confirmed in experiments with other soil-dwelling species, namely, *Burkholderia thailandensis*, *B. cenocepacia*, *B. stabilis*, *B. multivorans*, *B. cepacia*, and *Cupriavidus gilardii*, which did not show any growth in ACER medium (Supplementary Figure S7).

### Cultivation of soil samples in ACER medium resulted in the highest *Burkholderia pseudomallei* enrichment compared to TBSS-C50 and TBSS-C50-based erythritol medium

**Figure 6:**
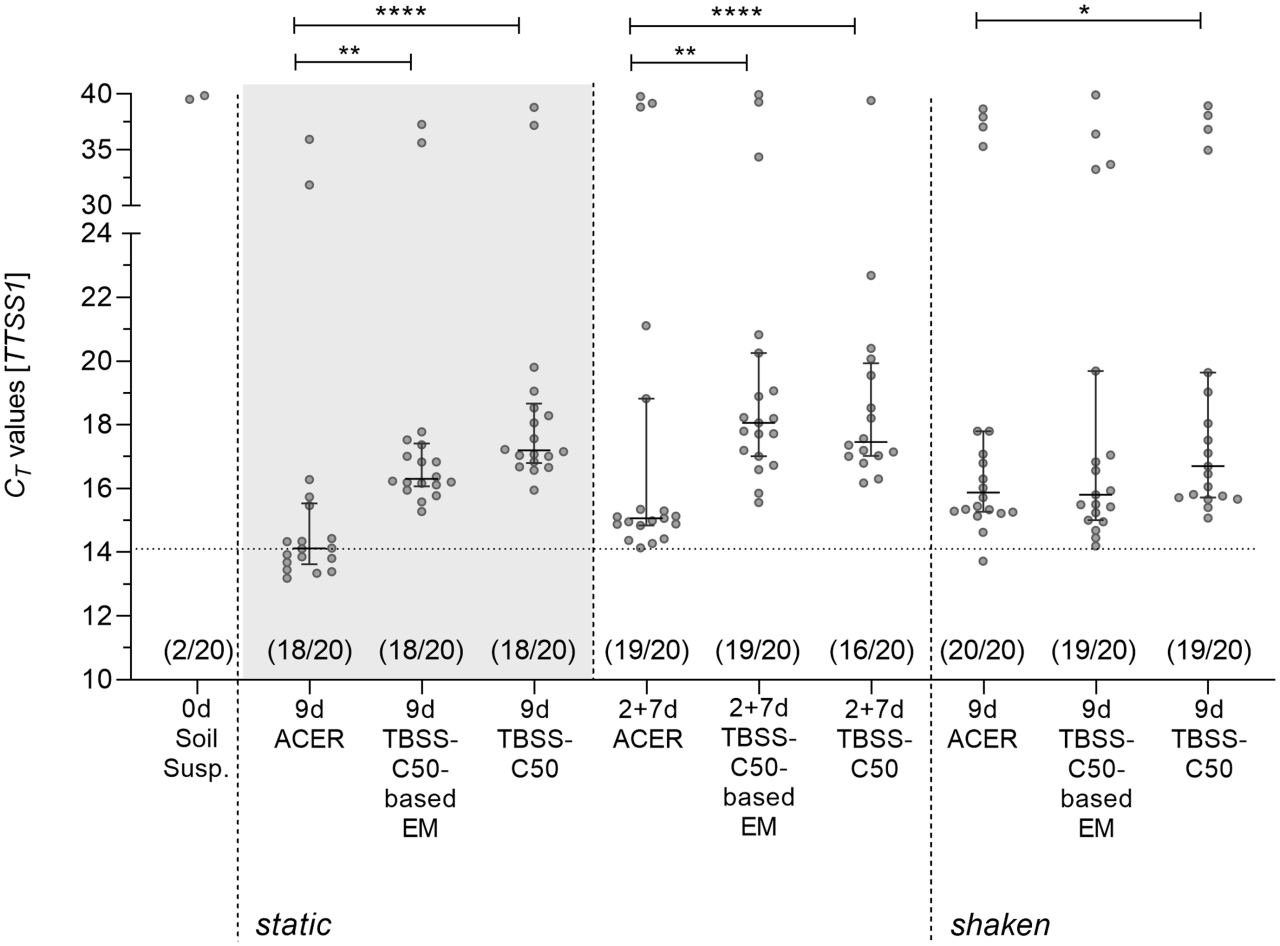
Molecular detection of *B. pseudomallei* in supernatants from soil sample enrichments in ACER medium, TBSS-C50-based erythritol medium (EM) and TBSS-C50. Twenty soil samples collected in October 2022 were subjected to different culture procedures with TBSS-C50, TBSS-C50-based erythritol medium and ACER medium as depicted in Figure 1. Quantitative PCR results of continuous cultures for 9  days with static and shaken incubation and for a static two-step culture of 2  days followed by 7  days are shown. The *C_T_* values with corresponding medians (horizontal line) and interquartile range are depicted in the graph. Each dot represents a single enrichment culture from a soil sample suspension performed in 10 mL medium. Samples highlighted within the grey box with *C_T_* values below 30 were plated on Ashdown agar (Figure 7). The total number of qPCR-positive samples for each culture protocol is shown below the corresponding data points above the abscissa (*p < 0.05, **p < 0.01, and ****p < 0.0001, “ns”, not significant; Friedman test with Dunn’s correction for paired data).

To finally test the newly developed ACER medium for its potential to increase the detection and recovery of *B. pseudomallei* from soil samples, we validated ACER medium in comparison with TBSS-C50-based erythritol medium and TBSS-C50 with twenty samples collected at the end of the wet season in October 2022 in the north-central part of Vietnam. Comparing 9 days continuous static culture of ACER medium (*C_T_* = 14.12; q1 = 13.62, q3 = 15.53) with TBSS-C50-based erythritol medium (*C_T_* = 16.30; q1 = 16.07, q3 = 17.41, *p* = 0.0002) and TBSS-C50 broth (*C_T_* = 17.19; q1 = 16.79, q3 = 18.66, *p* < 0.0001) by qPCR clearly revealed a superior enrichment of *B. pseudomallei* in ACER medium (Figure 6). Interestingly, the comparison of static and shaken 9 days ACER cultures (*C_T_* ^9d, shaken^ = 15.87; q1 = 15.27, q3 = 17.80) revealed no improved but a slightly lower enrichment of *B. pseudomallei* in the latter and a less clear advantage of ACER medium (Figure 6). We furthermore investigated the effect of a static ACER two-step culture with a transfer step after 2 days into new medium followed by 7 days of incubation (Figure 6). Again, we detected the highest enrichment with ACER medium (*C_T_* ^2 + 7d, static^ = 15.06; q1 = 14.84, q3 = 18.82) compared to TBSS-C50 (*C_T_* ^2 + 7d, static^ = 17.46; q1 = 17.02, q3 = 19.93, *p* < 0. 0001) and TBSS-C50 based erythritol medium (*C_T_* ^2 + 7d, static^ = 18.06; q1 = 17.01, q3 = 20.25, *p* = 0.003). However, in this setting, the two-step procedure was not superior to the continuous static enrichment for 9 days (Figure 6).

### Enrichment of soil samples in ACER medium significantly improves the recovery of viable *Burkholderia pseudomallei* on Ashdown agar plates

**Figure 7:**
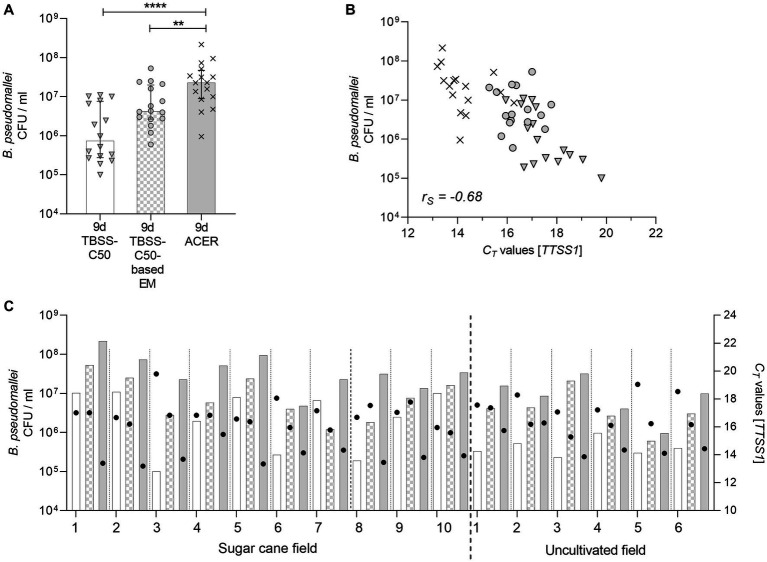
Effects of different enrichment media on the recovery of *B. pseudomallei* on Ashdown agar. **(A)** Twenty soil samples collected in October 2022 were incubated 9  days statically at 40°C in TBSS-C50, TBSS-C50-based erythritol medium (EM) and ACER medium. Sixteen soil samples with *C_T_* values below 30 in the three media after 9  days of static incubation were culture positive on Ashdown agar. Colony counts are log 10-transformed and depicted as bars (TBSS-C50 enrichments: plane bar; TBSS-C50-based erythritol medium: square patterned bar; ACER medium: grey bar), reflecting the median with interquartile range of all enrichments in the same medium. Recovered CFUs from single enrichments (means of duplicates cultured from each supernatant) are shown as symbols within the bars. The CFUs from TBSS-C50 are symbolized by grey triangles, from TBSS-C50-based erythritol medium by grey circles and from ACER medium by crosses (**p < 0.01 and ****p < 0.0001; Friedman test with Dunn’s correction for paired data). **(B)** The CFU values of all agar counts are plotted against their respective *C_T_* values. Again, grey triangles refer to CFU on Ashdown agar derived from TBSS-C50 enrichments, circles to TBSS-C50-based erythritol medium and crosses refer to ACER cultures. Spearman’s rank correlation was performed (“*r*_s_” Spearman’s rank correlation coefficient) to analyze the relationship between cultivated CFUs and respective *C_T_* values. **(C)** The graph shows CFU values depicted as bars (left *Y* axis) and corresponding *C_T_* values demonstrated as circles (right *Y* axis) after enrichment in all three media for every single soil sample (TBSS-C50 enrichments: plane bar; TBSS-C50-based erythritol medium: square patterned bar; ACER medium: grey bar).

We next cultivated qPCR positive soil enrichments from all three media after 9 days of static cultures on Ashdown agar (Figure 6, respective sample sets highlighted with a grey box). Sixteen soil samples with CT values below 30 in all three media were culture positive (Figures 7A,C). The remaining culture-negative samples were either PCR-negative or had CT values above 30 (Figure 6) of which the latter did not lead to the recovery o *B. pseudomallei* from agar. This is in accordance with our previous study, where no *B. pseudomallei* growth was obtained on Ashdown agar from any TBSS-C50 or TBSS-C50-based erythritol medium culture with *C_T_* values above 30 (Trinh et al., 2019) 30.7 times higher CFU counts were recovered from ACER enrichments (median CFU = 2.27 * 10^7^, q1 = 8.86 * 10^6^, q3 = 4. 70 * 10^7^) compared to TBSS-C50 (median CFU = 7.4 * 10^5^, q1 = 2.74 * 10^5^, q3 = 7.61 * 10^6^; *p* < 0.00001) and 5.4 times higher TBSS-C50-based erythritol medium cultures (median CFU = 4.2 * 10^6^, q1 = 2.68 * 10^6^, q3 = 1.98 * 10^7^; *p* = 0.005) (Figure 7A). The comparison of *B. pseudomallei* CFUs with the corresponding *C_T_* values from all enrichments (Figure 7B) revealed a strong negative correlation between the *C_T_* values and the recovery of *B. pseudomallei* CFUs on Ashdown agar (*r*_S_ = −0.68, *p* < 0.0001). Figure 7C depicts the recovery of viable cells from the three different culture media for single soil samples together with the corresponding *C_T_* values. In all 16 samples, ACER led to the highest CFU numbers of *B. pseudomallei* on agar plates. The increase in the recovery of viable cells from single samples cultivated in ACER medium compared to TBSS-C50-based erythritol medium ranged from 1.19 fold to 18.99 fold. The increase in ACER medium compared to TBSS-C50 ranged from 3.17 fold to 228 fold.

## Discussion

Important questions regarding the ecology and global prevalence of *B. pseudomallei* are still unanswered, not least, because of methodological difficulties when isolating this pathogen from environmental habitats. The cultivation of *B. pseudomallei*, especially from soil samples, is a major challenge, as the presence of numerous related bacteria with similar growth requirements and antibiotic resistance patterns may outcompete or inhibit its growth (Rhodes and Schweizer, 2016). However, isolating *B. pseudomallei* strains from their environmental reservoirs remains essential for the identification of potential risk areas for infection, for studies on *B. pseudomallei* virulence properties, antibiotic resistance and the bacterial population structure. Several studies have shown that *B. pseudomallei* can be detected by PCR methods directly in soil and soil enrichment cultures, from which it cannot be isolated on solid media (Brook et al., 1997; Trung et al., 2011; Lau et al., 2014; Knappik et al., 2015; Gohler et al., 2017; Trinh et al., 2019). Although molecular detection does not necessarily indicate the presence of viable and culturable bacteria, the high discrepancy between molecular and culture-based *B. pseudomallei* detection rates points to a remarkable lack of sensitivity of commonly applied culture methods. Moreover, the fact, that culture-based environmental screenings in regions of high melioidosis endemicity have resulted in either no or only very few culture-positive samples (Benoit et al., 2015; Wiersinga et al., 2015; Shaw et al., 2022), further indicates a potential sensitivity problem of the available culture methods used in those studies, including the currently recommended consensus method using TBSS-C50 (Limmathurotsakul et al., 2013). We recently accomplished a significant improvement in the isolation rate of *B. pseudomallei* from rice paddy soil samples by introducing TBSS-C50-based erythritol medium in which erythritol is the sole carbon source (Trinh et al., 2019). However, there was still a significant gap between the number of PCR-positive enrichment cultures with high *C_T_* values, indicating low bacterial concentrations, and the number of *B. pseudomallei*-positive subcultures on Ashdown agar from those samples (Trinh et al., 2019). Therefore, we aimed to optimize the erythritol-based culture conditions for *B. pseudomallei* to further improve the recovery of viable bacteria from soil samples.

As the pH has a major impact on bacterial growth and physiology (Olson, 1993), we, firstly, investigated the effect of the medium pH in erythritol medium. There is only a very small amount of information available on pH tolerance and preferences of *B. pseudomallei* in culture media (Dejsirilert et al., 1991). In a study dating back to 1991, Dejsirilert et al. described *B. pseudomallei* growth in highly nutritious heart infusion broth with an initial pH of 4.5 (Dejsirilert et al., 1991). Our experiments with erythritol as the sole carbon source showed that the lag phase of *B. pseudomallei* cultures in erythritol medium was significantly shortened at acidic pH values between 6.0 and 6.5, but growth stagnated at later time points. The culture growth stagnation was associated with a further decrease of medium pH of about 1.5 to 2 pH points, and, at the lowest, down to pH 4. We were able to stabilize the pH and thus growth at later time points and to maintain the earlier onset of growth by increasing the buffer capacity of the erythritol medium. The shortening of the lag phase we observed under acidic conditions, is consistent with several environmental as well as experimental studies that reported a high abundance of *B. pseudomallei* in slightly acidic habitats (Dejsirilert et al., 1991; Chen et al., 2003; Palasatien et al., 2008; Draper et al., 2010; Robertson et al., 2010; Wang-Ngarm et al., 2014; Musa et al., 2016). If the association with acidic environmental habitats is indeed the result of beneficial growth conditions and/or a higher resistance compared to competing microbes remains to be elucidated.

Another significant medium improvement was achieved by increasing the erythritol concentration in combination with the addition of vitamins, meanwhile, maintaining the selectivity of the broth (Figure 3; Supplementary Figure S7). Since most laboratories routinely apply static enrichment cultures, we aimed to improve *B. pseudomallei* growth under those oxygen-limited conditions by the addition of nitrate as a terminal electron acceptor for *B. pseudomallei* (Kraft et al., 2011; Mangalea et al., 2017). Indeed, the addition of nitrate increased the final optical densities of *B. pseudomallei* in static cultures (Figure 4; Supplementary Figure S3). The final medium composition of our new ACER medium, incorporating all amendments discussed above, led to a remarkably improved growth *of B. pseudomallei* pure cultures under static and shaken culture conditions compared to TBSS-C50 and TBSS-C50-based erythritol medium (Figure 5; Supplementary Figures S5, S6). Although we already included *B. pseudomallei* strains from a number of different Asian countries and from Africa, future studies will have to extend those experiments and confirm the growth advantages of *B. pseudomallei* in ACER medium with strains from other parts of the world such as Australia and the Americas. So far, we did not observe growth of non-*B. pseudomallei* species in ACER medium, although the existence of other soil bacteria capable of using erythritol as the sole carbon source under those conditions cannot be excluded.

The comparison of the three media for *B. pseudomallei* enrichment from soil samples showed that ACER medium not only improved growth of *B. pseudomallei* in pure culture, but indeed led to the highest enrichment of the bacterium from soil samples as determined by qPCR (Figure 6). Interestingly, in contrast to our growth experiments with isolated strains, shaken culture conditions did not lead to a higher enrichment of *B. pseudomallei* compared to static soil cultures after 9 days. One might speculate that static culture conditions are better suited to the metabolic state of soil embedded *B. pseudomallei* and, thereby, are initially better suited to promote growth. Since soil is a highly competitive environment (Waksman and Woodruff, 1940) and antagonistic bacteria of *B. pseudomallei* have been described (Marshall et al., 2010; Lin et al., 2011; Boottanun et al., 2017), it is also conceivable that the higher oxygen input in shaken cultures promotes survival and/or growth of *B. pseudomallei* antagonistic aerobic microbes which might still grow on soil-derived nutrients.

In our previous study, we observed an increased enrichment of *B. pseudomallei* from paddy soils in TBSS-C50-based erythritol medium compared to TBSS-C50 only by using a two-step culture, but not in a single step culture (Trinh et al., 2019). In the present study, the ACER one-step enrichment was not inferior to the ACER two-step approach, but even resulted in a slightly better enrichment under those experimental conditions (Figure 6). A possible explanation could be the lower primary soil inoculum of only 0.5 mL soil suspension in 10 mL medium in the present study compared to 10 g of soil in 20 mL in our previous study (Trinh et al., 2019). Under these conditions, the deposition of soil-derived components (e.g., additional carbon sources) affecting the selectivity of the erythritol-based medium was likely to be reduced, making a two-step culture unnecessary. Apart from the lower primary inoculum, it is also conceivable, that the improved formulation of the ACER medium itself might have contributed to the similar outcome of the single- and two-step procedure in our current study. Given the enormous variation of local soil characteristics, such as the *B. pseudomallei* load and microbial composition, nutrient content, and geochemical factors, the optimum for parameters such as the amount of the initial soil inoculum and the incubation time for the most efficient isolation of *B. pseudomallei*, will likely differ between endemic areas. The most important outcome of this study is that the superior enrichment in ACER medium led to the highest recovery of viable *B. pseudomallei* counts from single samples compared to TBSS-C50 and TBSS-C50-based erythritol medium (Figure 7). The wide range of fold increase in the recovery of viable cells from different samples with ACER medium is likely to be the result of a heterogeneity between single soil samples in terms of abiotic and biotic factors. Considerable differences in the presence of microorganisms, nutrients, water content etc. exist also in soil of very small volume (Vos et al., 2013). Studies are under way to validate the potential of our optimized medium to increase the sensitivity of *B. pseudomallei* isolation from non-sterile clinical samples in the diagnostic laboratory. All components of the ACER medium are chemically defined and widely commercially available. Taken together, we demonstrate that our highly selective ACER medium significantly improves *B. pseudomallei* enrichment and isolation from soil samples and has the potential to increase the rate of culture-positive *B. pseudomallei* samples in environmental screenings. This will not only facilitate isolation of viable cells for antibiotic resistance and virulence analyses but also help to further unravel the diversity of the *B. pseudomallei* population in soil samples (Roe et al., 2022).

## Data availability statement

The original contributions presented in the study are included in the article/Supplementary material, further inquiries can be directed to the corresponding author.

## Author contributions

KA and IS conceptualized the study, planned experiments, and analyzed the overall data and wrote the manuscript. TT planned and supervised soil sampling. KA, SL, LB, BM, DE, and AV performed experiments in the laboratory. KA drafted the figures and performed the statistical analysis. All authors contributed to the article and approved the submitted version.

## Conflict of interest

The authors declare that the research was conducted in the absence of any commercial or financial relationships that could be construed as a potential conflict of interest.

## Publisher’s note

All claims expressed in this article are solely those of the authors and do not necessarily represent those of their affiliated organizations, or those of the publisher, the editors and the reviewers. Any product that may be evaluated in this article, or claim that may be made by its manufacturer, is not guaranteed or endorsed by the publisher.
