# Supplementary material for: Rational design of an acidic erythritol (ACER) medium for the enhanced isolation of the environmental pathogen Burkholderia pseudomallei from soil samples
Source: Front Microbiol. 2023 Jun 30;14:1213818. doi: 10.3389/fmicb.2023.1213818 (PMC10353019; doi:10.3389/fmicb.2023.1213818)
Supplement: Supplementary file 1 [file Image_1.pdf]

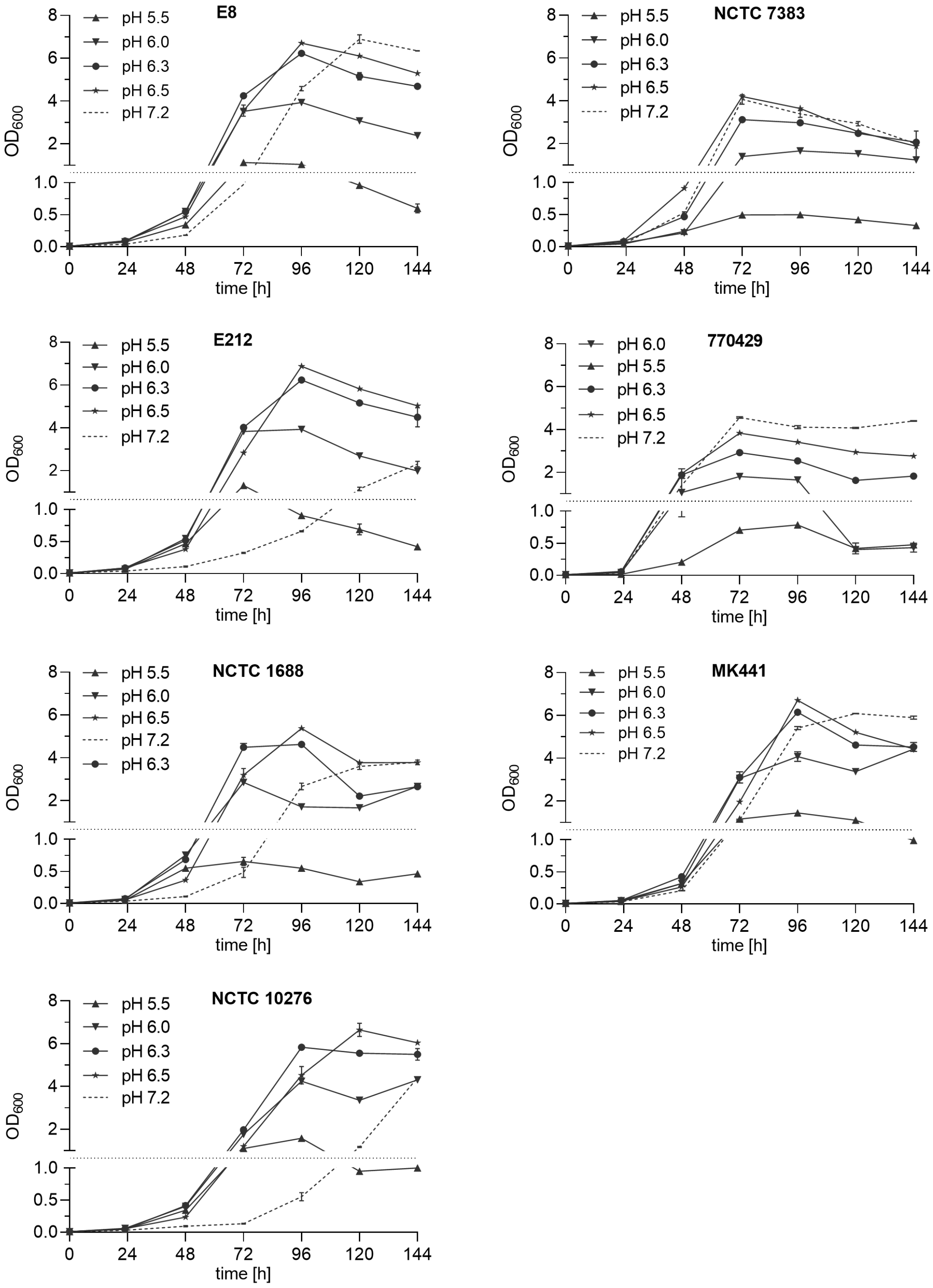
E8
pH 5.5
pH 6.0
pH 6.3
pH 6.5
pH 7.2
OD600
time [h]
NCTC 7383
pH 5.5
pH 6.0
pH 6.3
pH 6.5
pH 7.2
E212
pH 5.5
pH 6.0
pH 6.3
pH 6.5
pH 7.2
770429
pH 6.0
pH 5.5
pH 6.3
pH 6.5
pH 7.2
NCTC 1688
pH 5.5
pH 6.0
pH 6.5
pH 7.2
pH 6.3
MK441
pH 5.5
pH 6.0
pH 6.3
pH 6.5
pH 7.2
NCTC 10276
pH 5.5
pH 6.0
pH 6.3
pH 6.5
pH 7.2
0
24
48
72
96
120
144

Supp. Fig. 1. Impact of pH on the growth of *B. pseudomallei* strains in TBSS-C50-based erythritol medium. *B. pseudomallei* strains were cultured in 25 ml of TBSS-C50-based erythritol medium adjusted to pH 5.5, 6.0, 6.5 or 7.2 at 40 °C and 120 rpm. The growth was monitored by measuring OD_{600} every 24 h for up to 144 h (note the broken axis highlighted by a dashed line). Growth curves are representative of at least two independent experiments, each of which was conducted in technical duplicates. Error bars denote the standard deviation of mean from technical duplicates of a single experiment.
