# Supplementary material for: Rational design of an acidic erythritol (ACER) medium for the enhanced isolation of the environmental pathogen Burkholderia pseudomallei from soil samples
Source: Front Microbiol. 2023 Jun 30;14:1213818. doi: 10.3389/fmicb.2023.1213818 (PMC10353019; doi:10.3389/fmicb.2023.1213818)
Supplement: Supplementary file 2 [file Image_2.pdf]

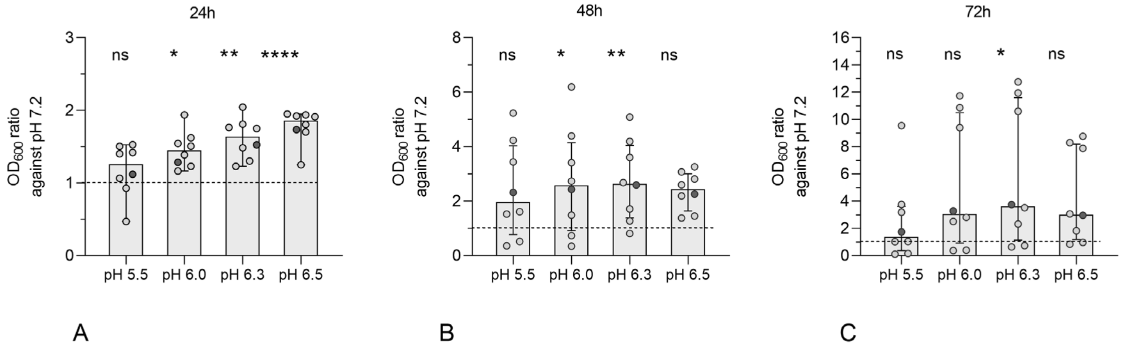

Supp. Fig. 2. Ratio of *B. pseudomallei* OD_{600} at different pH values against pH 7.2 in TBSS-C50-based erythritol medium at different time points.

The quotient of OD_{600} values of eight *B. pseudomallei* strains (listed in Table 1) grown in TBSS-C50-based erythritol medium of pH 5.5, 6.0, 6.3 and 6.5 and pH 7.2 after 24 h **(A),** 48 h **(B)** and 72 h **(C)**. The median of all strains with an interquartile range from two identical replicates is depicted. Values above the dashed line indicate higher optical densities at the respective time points compared to growth at pH 7.2. Circles represent data of single strains, the dark circle shows the quotient of strain K96243 (*$p < 0.05$; **$p < 0.01$; ****$p < 0.0001$; “ns” not significant; Friedman’s test with Dunn´s correction).
