# Supplementary material for: Rational design of an acidic erythritol (ACER) medium for the enhanced isolation of the environmental pathogen Burkholderia pseudomallei from soil samples
Source: Front Microbiol. 2023 Jun 30;14:1213818. doi: 10.3389/fmicb.2023.1213818 (PMC10353019; doi:10.3389/fmicb.2023.1213818)
Supplement: Supplementary file 3 [file Image_3.pdf]

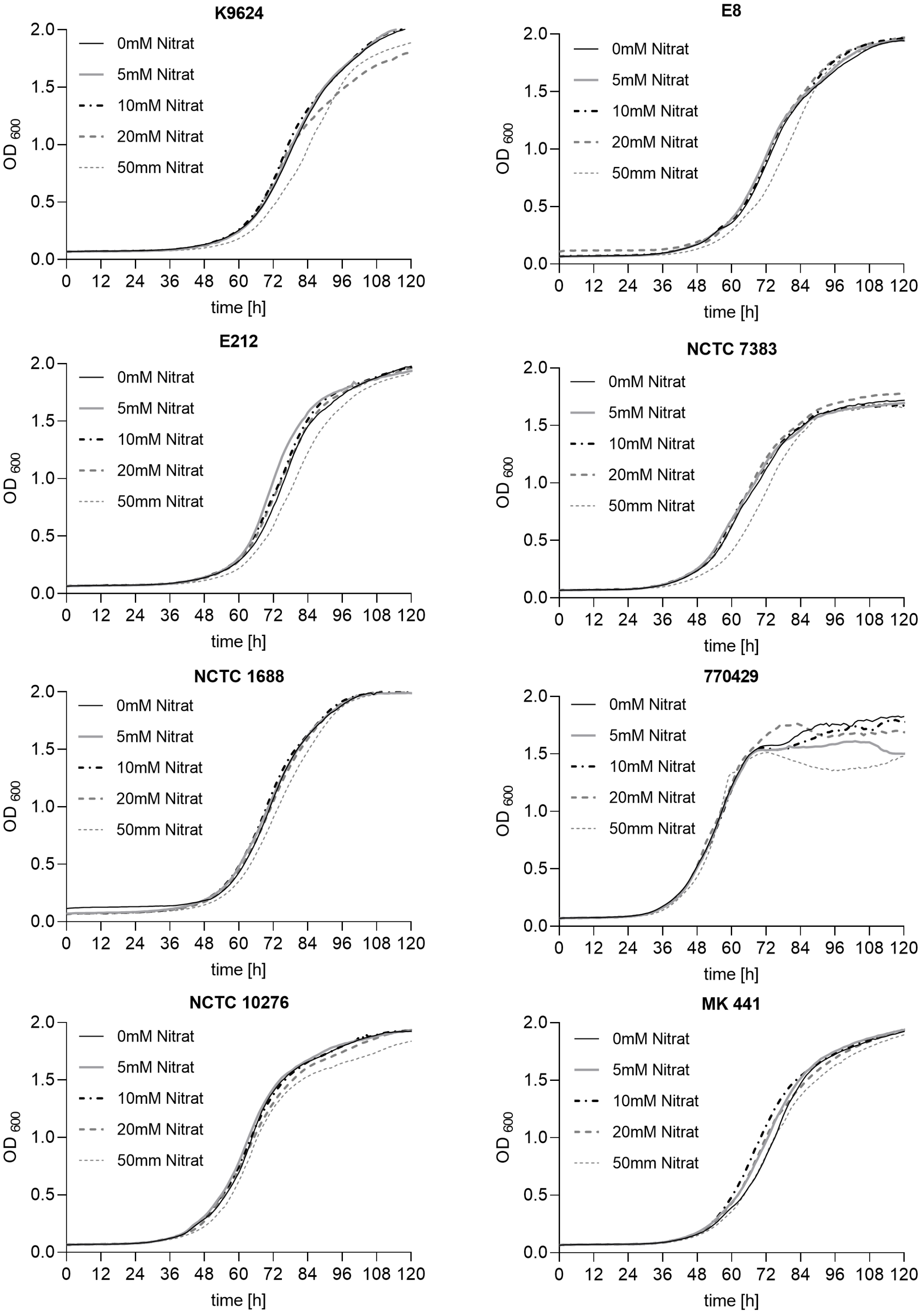

K9624
E8
E212
NCTC 7383
NCTC 1688
770429
NCTC 10276
MK 441
0mM Nitrat
5mM Nitrat
10mM Nitrat
20mM Nitrat
50mm Nitrat
OD 600
time [h]
0.0
0.5
1.0
1.5
2.0
0 12 24 36 48 60 72 84 96 108 120

Supp. Fig. 3. Effect of different nitrate concentrations on the growth of *B. pseudomallei* in modified TBSS-C50-based erythritol medium of pH 6.3, 50 mM potassium phosphate buffer, 1.2 % erythritol and Gibco MEM Vitamin Solution 1:50.

Eight *B. pseudomallei* strains were cultivated under continuous shaking with a medium amplitude for 144 h at 40 °C in 200 µl enrichment medium in a Bioscreen C instrument (Labsystems, Helsinki, Finland). The concentrations of sodium nitrate tested ranged from 0 to 50 mM. OD_{600} was monitored hourly. Strain names are shown in bold letters above the respective figure. Growth curves are representative of three independent experiments, each of which was conducted in technical duplicates. Error bars denote the standard deviation of mean from technical duplicates of a single experiment.
