# Supplementary material for: Rational design of an acidic erythritol (ACER) medium for the enhanced isolation of the environmental pathogen Burkholderia pseudomallei from soil samples
Source: Front Microbiol. 2023 Jun 30;14:1213818. doi: 10.3389/fmicb.2023.1213818 (PMC10353019; doi:10.3389/fmicb.2023.1213818)
Supplement: Supplementary file 4 [file Image_4.pdf]

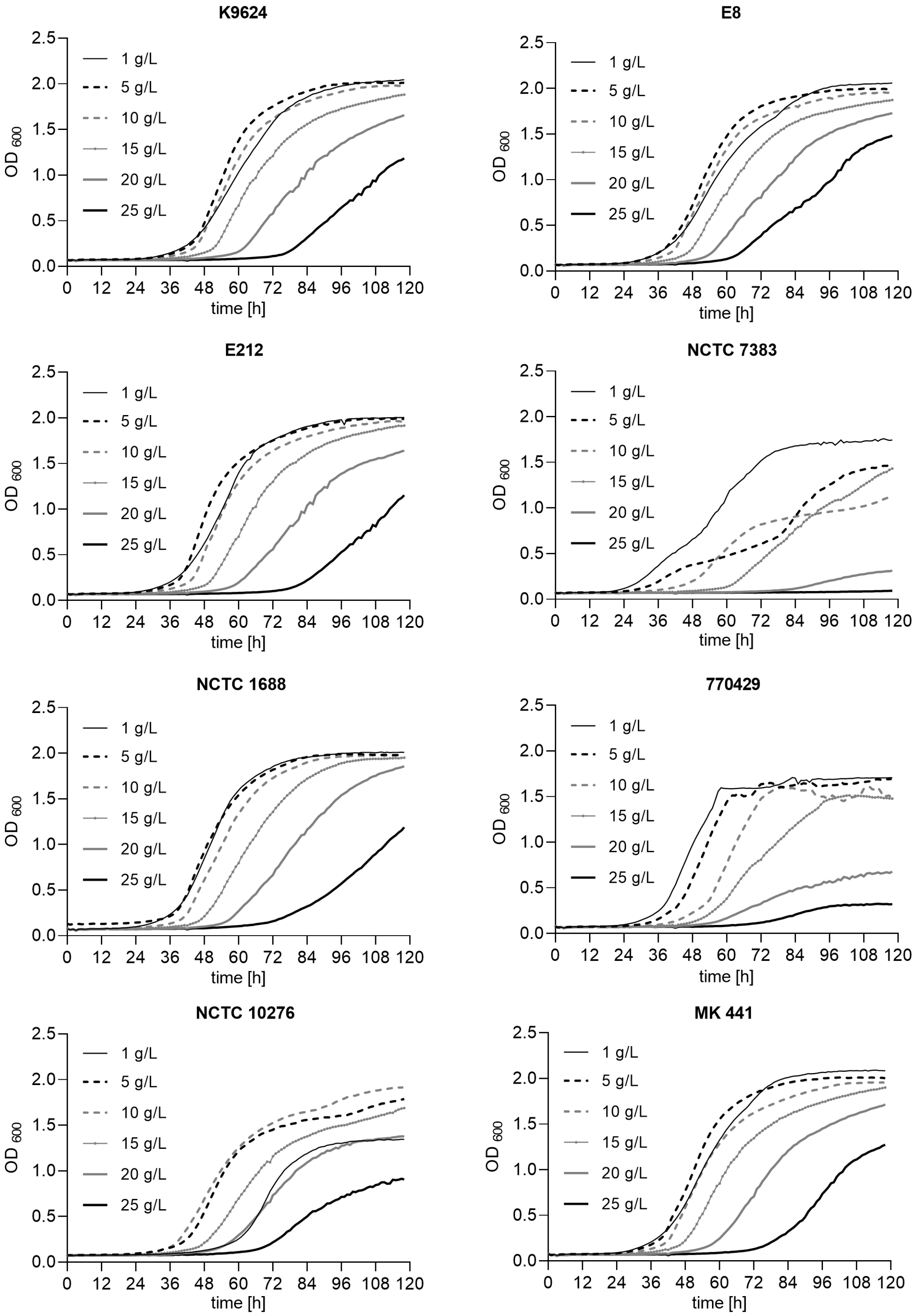
K9624
E8
E212
NCTC 7383
NCTC 1688
770429
NCTC 10276
MK 441
1 g/L
5 g/L
10 g/L
15 g/L
20 g/L
25 g/L
OD 600
time [h]
0 12 24 36 48 60 72 84 96 108 120
0.0 0.5 1.0 1.5 2.0 2.5

Supp. Fig. 4. Impact of sodium chloride concentrations on the growth of *B. pseudomallei* in modified TBSS-C50-based erythritol medium of pH 6.3, 50 mM potassium phosphate buffer concentration, 1.2 % erythritol, Gibco MEM Vitamin Solution 1:50 and 10mM nitrate.

Eight *B. pseudomallei* strains were cultivated under continuous shaking with a medium amplitude for 144 h at 40 °C in 200 µl medium in a Bioscreen C instrument (Labsystems, Helsinki, Finland). The concentrations of sodium chloride tested ranged from 0.1 to 2.5 %. OD_{600} was monitored hourly. Strain names are shown in bold letters above the respective growth curves. Growth curves are representative of two independent experiments, each of which was conducted in technical duplicates. Error bars denote the standard deviation of mean from technical duplicates of a single experiment.
