# Supplementary material for: Rational design of an acidic erythritol (ACER) medium for the enhanced isolation of the environmental pathogen Burkholderia pseudomallei from soil samples
Source: Front Microbiol. 2023 Jun 30;14:1213818. doi: 10.3389/fmicb.2023.1213818 (PMC10353019; doi:10.3389/fmicb.2023.1213818)
Supplement: Supplementary file 6 [file Image_6.pdf]

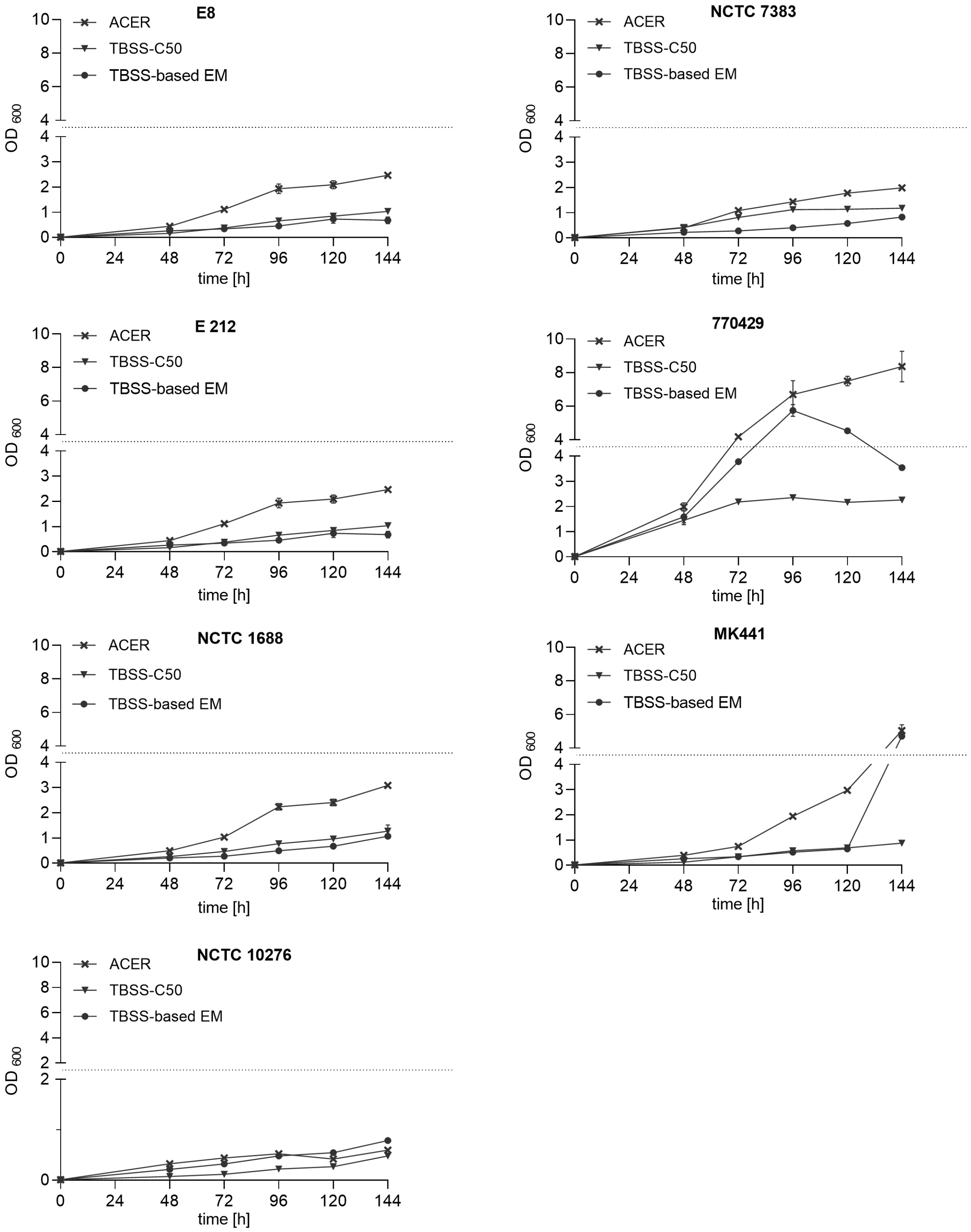
E8
NCTC 7383
E 212
770429
NCTC 1688
MK441
NCTC 10276
ACER
TBSS-C50
TBSS-based EM
OD 600
time [h]
0
24
48
72
96
120
144

Supp. Fig. 6. Static incubation of *B. pseudomallei* in optimized ACER medium compared to TBSS-C50 and TBSS-C50-based erythritol medium.

Eight different *B. pseudomallei* strains were cultivated statically with loosely screwed 50 ml falcons in a volume of 10 ml for 144 h at 40 °C in ACER medium, TBSS-C50 and TBSS-C50-based erythritol medium. The optical density at 600 nm was measured every 24 h starting from 48 h onward (note the broken axis highlighted by a dashed line). Strain names are shown in bold letters above the respective figure. Growth curves are representative of at least two independent experiments, each experiment was conducted in technical duplicates. Error bars denote the standard deviation of mean from technical duplicates of a single experiment.
