# Supplementary material for: Rational design of an acidic erythritol (ACER) medium for the enhanced isolation of the environmental pathogen Burkholderia pseudomallei from soil samples
Source: Front Microbiol. 2023 Jun 30;14:1213818. doi: 10.3389/fmicb.2023.1213818 (PMC10353019; doi:10.3389/fmicb.2023.1213818)
Supplement: Supplementary file 7 [file Image_7.pdf]

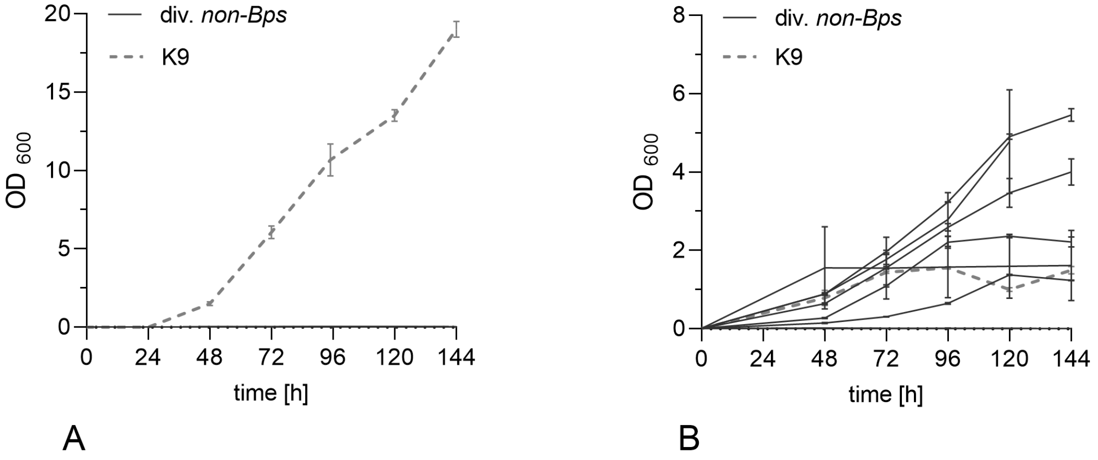

Supp. Fig. 7. Selectivity of ACER medium compared to TBSS-C50.

B. pseudomallei strain K9624 and different isolates of non-*B. pseudomallei strains*, *B. thailandensis*, *B. cenocepacia, B. stabilis, B. multivorans, B. cepacia* and *Cupriavidus gilardii* were cultivated at 120 rpm in 50 ml falcons in 10 ml medium at 40 °C. OD_{600} was measured every 24 h starting from 48 h onward. Growth curves of *B. pseudomallei* strain K9624 are highlighted with a dotted line, while growth curves of all non-*B. pseudomallei* isolates are shown as solid lines. **(A)** Growth kinetics of isolates tested in ACER medium without the addition of colistin **(B)** Growth kinetics of isolates tested in TBSS-C50. Growth curves are representative of two independent experiments, each of which was conducted in technical duplicates. Error bars denote the standard deviation of mean from technical duplicates of a single experiment.
